# Modelling the emergence of influenza drug resistance: The roles of surface proteins, the immune response and antiviral mechanisms

**DOI:** 10.1371/journal.pone.0180582

**Published:** 2017-07-10

**Authors:** Hana M. Dobrovolny, Catherine A. A. Beauchemin

**Affiliations:** 1 Department of Physics & Astronomy, Texas Christian University, Fort Worth, TX, United States of America; 2 Department of Physics, Ryerson University, Toronto, ON, Canada; 3 Interdisciplinary Theoretical Science (iTHES) Research Group at RIKEN, Wako, Japan; Universidad Autonoma de Madrid Centro de Biologia Molecular Severo Ochoa, SPAIN

## Abstract

The emergence of influenza drug resistance has become of particular interest as current planning for an influenza pandemic involves using massive amounts of antiviral drugs. We use semi-stochastic simulations to examine the emergence of drug resistant mutants during the course of a single infection within a patient in the presence and absence of antiviral therapy. We specifically examine three factors and their effect on the emergence of drug-resistant mutants: antiviral mechanism, the immune response, and surface proteins. We find that adamantanes, because they act at the start of the replication cycle to prevent infection, are less likely to produce drug-resistant mutants than NAIs, which act at the end of the replication cycle. A mismatch between surface proteins and internal RNA results in drug-resistant mutants being less likely to emerge, and emerging later in the infection because the mismatch gives antivirals a second chance to prevent propagation of the mutation. The immune response subdues slow growing infections, further reducing the probability that a drug resistant mutant will emerge and yield a drug-resistant infection. These findings improve our understanding of the factors that contribute to the emergence of drug resistance during the course of a single influenza infection.

## Introduction

The annual cost of influenza illness and the ongoing threat of emergence of a pandemic strain make it all the more necessary to revisit the treatment options currently available. Two classes of drugs, adamantanes and neuraminidase inhibitors (NAIs), are currently available for treatment of influenza, although resistance to both classes of drugs threatens our ability to effectively treat influenza [1]. Better understanding the processes underlying the emergence of drug resistance over the course of an influenza infection will enable health authorities to make more effective use of antivirals on a seasonal basis, or in the context of a pandemic.

The adamantanes and NAIs exert their antiviral effects by blocking different phases of the viral replication cycle. Adamantanes, such as rimantadine and amantadine, act at the start of the replication cycle by blocking the ion channel activity of the matrix M2 protein and preventing viral uncoating, and thus viral replication [2]. When used prophylactically, the effectiveness of adamantanes against influenza A is 61% [3], and their use can reduce the duration of illness by 1.5 days when treatment is initiated within 48 h of symptom onset [4]. NAIs, such as oseltamivir and zanamivir, act at the end of the replication cycle by blocking the activity of the neuraminidase (NA) protein which is responsible for the removal of sialic acids from oligosaccharides binding newly produced virus to the surface of the producing cells. By blocking NA activity, NAIs significantly hinder the ability of newly produced virions (virus particles) to free themselves from the bounds of the producing cells and the mucins, hence curbing or stopping further infection [5, 6]. The efficacy of oseltamivir and zanamivir in preventing influenza ranges from 58% to 84% [7], and both can reduce the duration of viral shedding in treated patients by 2–3 days [8, 9].

Resistance to adamantanes emerges rapidly during treatment [10–12] and experiments revealed that natural resistance to these drugs has been increasing [4, 13]. In fact, the fraction of influenza A/H3N2 virus harboring the S31N adamantane-resistant mutation in the United States increased from 1.9% in 2004 to 92.3% by the early 2005–2006 influenza season [4] and virtually all circulating influenza strains now harbor mutations conferring adamantane resistance. NAI resistance was thought to develop more slowly than resistance to adamantanes and virus harboring NAI-resistance are much less frequent, around 0.4%–1% in adults [14]. Oseltamivir resistance rose quite dramatically during the early 2008–2009 flu season dramatically, being detected in 98–100% of infections [15, 16]. However, unlike amantadine for which annual resistance, once it emerged, has remained high, the annual level of resistance to oseltamivir dropped again because the pandemic strain of H1N1 that swept around the world in 2009 is susceptible to oseltamivir [17]. Some of the variability in the level of resistance against oseltamivir is probably due to the fact that the main mutation conferring resistance against oseltamivir, the H275Y mutation in the N1 neuraminidase [18–20], can have a variety of effects on the viral life cycle [21, 22], sometimes causing little change in viral fitness [23, 24], other times resulting in an important loss of fitness of the strain carrying that mutation [25, 26], unless it also carries compensatory mutation [27].

Previous work on the emergence of drug-resistance in influenza A has focussed largely on epidemiological models [28–37] which describe the spread of drug-resistant infections across a population. While such studies are important for developing strategies to prevent the spread of drug resistance once it emerges, they do not provide insight into how the drug resistant mutant arises during the course of a single infection, and on what timescale. One early modelling study examined the emergence of drug resistance to NAIs during a single infection [38] finding that NAI-resistance could emerge in the absence of drug treatment, albeit at low levels, even if the mutant is slightly less fit than the wild-type virus. Several studies have used models to quantify the fitness difference caused by drug resistant mutations [21, 22, 39–41]. Other studies have used models to optimize treatment regimens to reduce the emergence of drug resistant mutants [42–44]. However, no study has yet tried to examine some of the biological processes that might help or hinder the emergence of drug resistance.

In this paper, the effect of treatment with adamantanes or NAIs on the emergence of drug resistant mutants is explored using several within-host mathematical models. The models contain several novel features; they allow for the possibility of back mutation (a mutant strain reverting back to its wild-type form) and they consider the effect of a mismatch between the viral surface proteins and the RNA contained within the virus. These models will be used to determine whether internal/external mismatch, antiviral mechanism, or the immune response affect how quickly drug resistance emerges during the course of a single infection.

## Materials and methods

### Establishing the rate of mutation

We are interested in mutations which produce drug resistance. Hence, we define a mutant as a strain which contains a nucleotide mutation at a specific position within one of the codons that encode the amino acid positions where drug-resistant mutations have been identified. The average mutation rate for influenza A is *μ*_nt_ = 7.3 × 10^−5^ per nucleotide per replication [45]. Although several drug-resistant mutations have been found for adamantanes [2, 4, 46, 47] and NAIs [6, 14], we will focus on only the most common drug mutations (S31N in the M2 protein for amantadine and H275Y in the N1 protein for oseltamivir, for example) and assume that they occur at the average mutation rate for influenza A. This assumes that a mutation anywhere other than at the specific nucleotide position within our specific codon does not lead to significant drug-resistance and does not affect viral fitness. Analogously, since a drug-resistant mutant can only revert to wild-type if there is a mutation at that same specific nucleotide which conferred it its resistance, we set *ρ*_*μ*→wt_ = *μ*_nt_. Note that a single mutation event during replication can give rise to production of many mutant virions since the mutated RNA can be copied once it appears. Hence, we can express *ρ*_*j*→*i*_, the probability with which a cell infected with viral strain *j* will produce a virion of strain *i*, as a simple function of the mutation rate per nucleotide per replication, *μ*_nt_, namely

$$\rho_{j\to i}=\left(\begin{array}{cc} 1-\mu_{\text{nt}} & \mu_{\text{nt}}\\ \mu_{\text{nt}} & 1-\mu_{\text{nt}} \end{array}\right).$$

### Model for early mutation events

**Fig 1:**
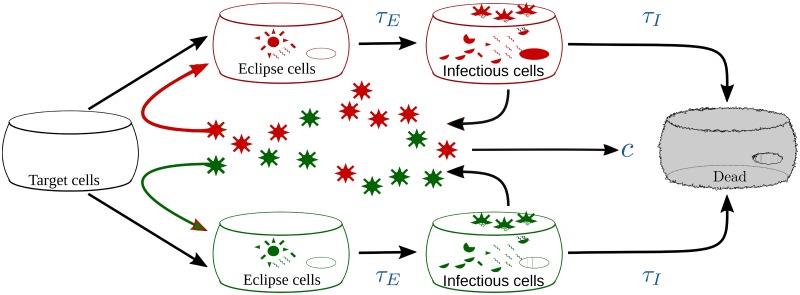
Drug resistance model. A mathematical model of influenza infections consisting of two viral strains: a wild-type and a drug resistant mutant.

We will be using a modified version of the model proposed in [48] with two viral subpopulations or strains: a wild-type, drug sensitive population, and a mutant, drug resistant population. The model is depicted graphically in Fig 1, and can be expressed as

$$\begin{array}{l} \frac{\text{d}T}{\text{d}t}=-T\sum_{i}\beta_{i}V_{i}\\ \frac{\text{d}E_{i}}{\text{d}t}=\left(1-m_{i}\right)\beta_{i}TV_{i}-\frac{E_{i}}{\tau_{E}}\\ \frac{\text{d}I_{i}}{\text{d}t}=\frac{E_{i}}{\tau_{E}}-\frac{I_{i}}{\tau_{I}}\\ \frac{\text{d}V_{i}}{\text{d}t}=\left(1-n_{i}\right)p_{i}\sum_{j}\rho_{ij}\;I_{j}-cV_{i}, \end{array}$$ (1)

where *T* is the pool of uninfected, susceptible target cells. When a target cell is infected, it enters the eclipse phase, *E*_*i*_, i.e., the cell is infected with viral strain *i* but is not yet producing virions. Cells remain in the eclipse phase for an average time *τ*_*E*_ before becoming infectious cells, *I*_*i*_. Infectious cells, *I*_*i*_, are cells productively infected with viral strain *i*, releasing virions of strain *i* at constant rate *p*_*i*_ with probability *ρ*_*i*→*i*_, and of strain *j* at constant rate *p*_*j*_ with probability *ρ*_*ij*_, where *ρ*_*ij*_ are the elements of *ρ*_*j*→*i*_ as described in Section. Infectious cells die after producing virus continuously for an average time *τ*_*I*_. While this is a target cell limited model, due to the short duration of influenza infection and the long regeneration time for cells in the respiratory tract [49], regenerated cells are not likely to play much of a role in infection dynamics. This model will be referred to as the complete mutation (CM) model.

Adamantanes prevent uncoating of the virus, thus blocking the infection, so their effect in our model is to reduce the infection rate *β*_*i*_. Note that we assume a cell cannot be infected by multiple virions, so we apply the effect of the drug only to *β*_*i*_ in the equations for eclipse cells, as described in [10]. In this formulation, target cells disappear at the usual rate, but these cells do not all appear in the eclipse phase, so some cells are effectively removed from the system. The biological interpretation of this is that since amantadine does not block cell entry, but rather blocks uncoating of the virus [2], virions will enter the cells at the same rate in the presence or absence of amantadine. At some point, however, cells will not be able to absorb any more virus, so will no longer be target cells, but since none of the absorbed virions are uncoated, these cells never enter the eclipse phase. NAIs prevent release of newly formed virions, so their effect is modelled as reducing the virus production rate *p*_*i*_ of virus strain *i*. The efficacies of adamantanes and NAIs at blocking viral strain *i* are *m*_*i*_ and *n*_*i*_, respectively. In modelling antiviral therapy, we set and hold *m*_*i*_ and *n*_*i*_ at a fixed value which means we assume that drug concentration increases instantaneously to its chosen value and remains constant for the duration of the infection.

Some parameters were assumed not to depend on the viral strain. Those are *τ*_*E*_, the duration of the eclipse phase; *τ*_*I*_, the infectious cell lifespan; and *c*, the rate at which virions are cleared by non-specific clearance (e.g. mucus) and immune responses. The values for these parameters were taken from [48] (see Table 1). Antiviral efficacy against resistant strains was chosen to be 0.005 since antiviral efficacy of resistant strains is known to be 10–10,000-fold less than against sensitive strains [50, 51]. Four parameters were chosen to depend on the viral strain. Those are *β*_*i*_, the rate of infection of target cells per concentration of virus of strain *i*; *p*_*i*_, the production rate of new virions of strain *i*; and *n*_*i*_ and *m*_*i*_, the efficacies of adamantanes and NAIs on viral strain *i*. For simplicity, we assume that a mutation conferring adamantane resistance can lead to a change in infection rate (i.e., *β*_*μ*_ ≠ *β*_wt_), but will not affect the production rate. Similarly, we assume a mutation conferring NAI resistance can lead to a change in production rate (i.e., *p*_*μ*_ ≠ *p*_wt_), but will not affect infection rate. In reality, NAI-drug resistant mutations could possibly leave the mutant virus more “sticky”, i.e. with a reduced ability to cleave the haemagglutinin (HA) receptor and free itself from the surface of the producing cell [5, 26, 51, 52] and a “stickier” virus is likely to be more capable of infecting a cell. In fact, the H275Y mutation is known to alter several different parameters, although which parameters are affected appears to depend on the genetic background in which the mutation is introduced [21, 22, 39]. However, this simplifying assumption allows us to easily quantify the fitness of a mutant strain; we define the *relative fitness* of the mutant compared to its wild-type counterpart as *β*_*μ*_/*β*_wt_ for mutations conferring resistance to adamantanes, or *p*_*μ*_/*p*_wt_ for those conferring resistance to NAIs. While other experimentally-derived quantities are used to assess relative fitness of two viruses [53, 54], since we know the underlying mechanism causing the fitness difference in this case, it seems natural to use it to quantify fitness. A similar definition of relative fitness has been used in previous studies [40, 41].

**Table 1:** Default initial conditions and parameter values for models. Unless otherwise indicated, values are taken from [48].

Symbol	Value
*T*(0)	4.0 × 10^8^ cells
*E*_*i*_(0) = *I*_*i*_(0)	0
*V*(0) = *V*_*μ*_(0)+*V*_wt_(0)	7.5 × 10^−2^ TCID_50_/mL
*τ*_*E*_	6.0 h
*τ*_*I*_	4.6 h
*c*	5.2 d^−1^
*β*_wt_	3.2 × 10^−5^ (TCID_50_/mL)^−1^ × d^−1^
*p*_wt_	4.6 × 10^−2^ TCID_50_/mL × d^−1^
*ρ*_*μ*wt_, *ρ*_wt*μ*_	7.3 × 10^−5^ [45]
*m*_wt_, *n*_wt_	0.6–0.98
*n*_*μ*_, *m*_*μ*_	0.005^1^

^1^Antiviral efficacy against resistant strains is 10–10,000-fold less than against sensitive strains.

### Models for late mutation events

**Fig 2:**
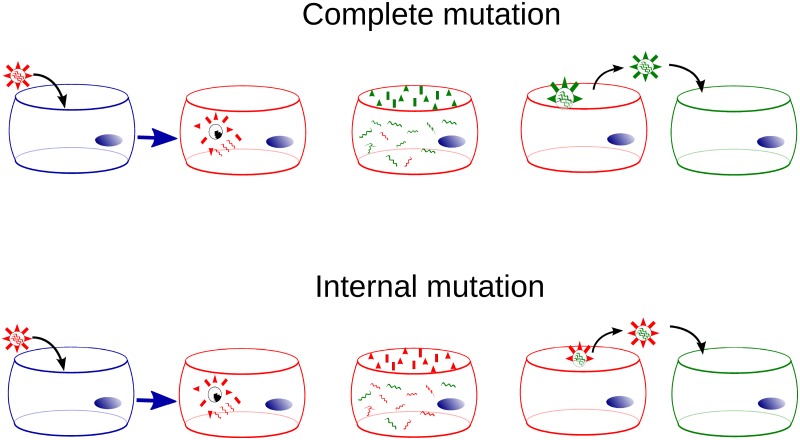
Mutation models. The complete mutation model assumes the mutation occurs before surface proteins are produced such that it is carried by both the surface and core protein. The internal mutation model assumes the mutation occurs after surface proteins have been produced such that it appears only in the internal, core protein, not in the surface protein. Cells infected by a virion containing a mutated core protein, irrespective of its surface proteins, will produce virus containing the mutation in both the core and surface protein.

Previous models of resistance emergence in vivo (our model (1), and that proposed in [38]), assume that when, for example, the H275Y mutation conferring resistance to oseltamivir occurs stochastically during viral replication within an infected cell, both the external NA expressed on the surface of the virus, and the internal, core NA RNA segment packed within the virion’s capsid will carry this mutation. This might not be the case. Studies have found that surface proteins appear on the apical surface of infected cells soon after infection: HA appears on the surface in 20–40 min [55, 56], NA in 1–3 h [57, 58], with M2 being the slowest, appearing on the cell’s surface 6–8 h after infection [59]. Synthesis of influenza viral RNA peaks at 5–6 h [60, 61], some time after the surface proteins are synthesized, and remains high over the remainder of the infected cell’s lifespan. If a mutation occurs early in the replication process, before the surface proteins have been produced, both the internal proteins and most of the surface proteins would contain that mutation (complete mutation). If, however, the mutation occurs later in the process, most surface proteins will not contain the mutation, but the internal core protein in these virions will (internal mutation). These two different models for antiviral resistance emergence are depicted in Fig 2.

In the complete mutation model (model (1) in Methods), the mutation occurs early in the replication process such that the core and surface viral proteins match (either both carry or neither carries the mutation). In the internal mutation models (models (2) and (3) in Methods), the mutation occurs after surface proteins have been produced, such that the core and surface viral proteins will not match: only the former will carry the mutation. In reality, there will probably be a mixture of wild-type and mutated surface proteins arising from a mutation. Our models allow us to investigate the two extreme cases which will help determine whether this variation plays a significant role in the emergence of drug resistance. In both the complete and internal models, once a virion carrying a mutation (either only in its core or both in its core and surface protein) infects another cell, this secondary cell will primarily produce virions carrying the mutation both in their core and surface proteins.

The different mode of action of adamantanes and NAIs require that a different late mutation model be developed for each drug class. For NAIs, the model equations remain largely unchanged from Eq (1), namely

$$\begin{array}{l} \frac{\text{d}T}{\text{d}t}=-\beta_{\text{wt}}T\left(V_{\text{wt}}+V_{\mu }\right)\\ \frac{\text{d}E_{i}}{\text{d}t}=\beta_{\text{wt}}TV_{i}-\frac{E_{i}}{\tau_{E}}\\ \frac{\text{d}I_{i}}{\text{d}t}=\frac{E_{i}}{\tau_{E}}-\frac{I_{i}}{\tau_{I}}\\ \frac{\text{d}V_{i}}{\text{d}t}=\sum_{j}\left(1-n_{i}\right)p_{j}\rho_{ij}\;I_{j}-cV_{i}, \end{array}$$ (2)

where only the equation for virus has changed (*n*_*i*_ → *n*_*j*_ and *p*_*i*_ → *p*_*j*_), so that a virion’s production rate and susceptibility to NAIs will depend on its external, surface NA (*j*) irrespective of its internal, core NA (*i*). Note that since we assume mutations conferring resistance to NAIs do not affect *β*, all virus types will have the same susceptibility to infection regardless of their core or surface proteins.

For adamantanes, the model is slightly different since we now assume that mutations in M2 conferring adamantane resistance will change *β*, but not *p*, such that

$$\begin{array}{l} \frac{\text{d}T}{\text{d}t}=-\beta_{\text{wt}}T\left[V_{\text{wt}}^{\text{wt}}+V_{\mu }^{\text{wt}}\right]-\beta_{\mu }T\left[V_{\text{wt}}^{\mu }+V_{\mu }^{\mu }\right]\\ \frac{\text{d}E_{i}}{\text{d}t}=T\sum_{j}\left(1-m_{j}\right)\beta_{j}V_{i}^{j}-\frac{E_{i}}{\tau_{E}}\\ \frac{\text{d}I_{i}}{\text{d}t}=\frac{E_{i}}{\tau_{E}}-\frac{I_{i}}{\tau_{I}}\\ \frac{\text{d}V_{i}}{\text{d}t}=p_{wt}\rho_{ij}\;I_{j}-cV_{i}^{j}, \end{array}$$ (3)

where $V_{i}^{j}$ corresponds to virions with internal core M2 of type *i*, and external surface M2 of type *j*. We therefore have 4 viral subpopulations whose infectivity and susceptibility to adamantanes depend on their surface M2, *j*, but whose viral progeny will have surface M2 of type *i*, and internal M2 predominantly of type *i*. These models will be referred to as the internal mutation (IM) models.

### Models with an immune response

We investigate the effects of an immune response by adding a simplified antibody response to our models. Since we are really only interested in how the immune response will stop the emergence of a drug-resistant mutant, we consider the growth of antibodies, but neglect the decay, as suggested by [38]. While not entirely realistic, we must consider whether the details we are neglecting are likely to substantially alter the dynamics we observe. Antibodies appear at around 5 dpi for primary infections and around 3 dpi for secondary infections [62]. The number of antibodies grows quickly eventually reaching a plateau around 10–12 dpi [63]. While the exponential growth assumed by our model reproduces the growth phase of antibodies fairly well, it is clearly incorrect once the antibodies plateau. Influenza infections are largely resolved by 7–8 dpi and during that time frame, the exponential growth of antibodies is a reasonable approximation. We modify all models with the addition of an equation describing the growth of antibodies and a slight modification to the equations describing virus. For example, the complete mutation model becomes

$$\begin{array}{l} \frac{\text{d}T}{\text{d}t}=-T\sum_{i}\beta_{i}V_{i}\\ \frac{\text{d}E_{i}}{\text{d}t}=\left(1-m_{j}\right)\beta_{i}TV_{i}-\frac{E_{i}}{\tau_{E}}\\ \frac{\text{d}I_{i}}{\text{d}t}=\frac{E_{i}}{\tau_{E}}-\frac{I_{i}}{\tau_{I}}\\ \frac{\text{d}X}{\text{d}t}=aX\\ \frac{\text{d}V_{i}}{\text{d}t}=\left(1-n_{i}\right)p_{i}\sum_{j}\rho_{ij}\;I_{j}-cV_{i}-k_{v}XV_{i}, \end{array}$$ (4)

where we have added a growth equation for antibodies, *X*, and an antibody-virus binding term in the equation for virus. We set the growth rate of antibodies, *a* = 1 d^−1^, the binding rate, *k*_*v*_ = 1 d^−1^ and the initial number of antibodies *X*_0_ = 0.34, as in [38]. Note that we assume that antibodies have an equal affinity and avidity for both the wild-type and mutant strains. Since our model assumes that wild-type and drug resistant viruses are identical except for the single mutation causing drug resistance. As such, the antigens on the two virus strains should be identical and therefore the antibody binding rate should be the same for both.

### Basic reproductive number

The basic reproductive number, *R*_0_, is the number of cells infected when a single infected cell is placed within a homogeneous susceptible population. Interestingly, the basic reproductive number is the same for both the CM and IM assumptions. *R*_0_ can be determined through a stability analysis of the differential equations of our models. For our models, the basic reproductive number is given by

$$\begin{array}{ccc} R_{0} & = & \frac{\left(1-m_{\mu }\right)\left(1-m_{\text{wt}}\right)\left(1-n_{\mu }\right)\left(1-n_{\text{wt}}\right)\beta_{\mu }\beta_{\text{wt}}p_{\mu }p_{\text{wt}}T_{0}^{2}\text{det}\left(\rho_{j\to i}\right)}{c^{2}\delta^{2}}\\ & - & \frac{\left(1-m_{\mu }\right)\left(1-n_{\mu }\right)\beta_{\mu }p_{\mu }\rho_{\mu \mu }T_{0}}{c\delta }-\frac{\left(1-m_{\text{wt}}\right)\left(1-n_{\text{wt}}\right)\beta_{\text{wt}}p_{\text{wt}}\rho_{\text{wtwt}}T_{0}}{c\delta }, \end{array}$$

where det(*ρ*_*j*→*i*_) is the determinant of *ρ*_*j*→*i*_.

For the models with an immune response, the basic reproductive number is modified by the immune response and becomes

$$\begin{array}{ccc} R_{0} & = & \frac{\left(1-m_{\mu }\right)\left(1-m_{\text{wt}}\right)\left(1-n_{\mu }\right)\left(1-n_{\text{wt}}\right)\beta_{\mu }\beta_{\text{wt}}p_{\mu }p_{\text{wt}}T_{0}^{2}\text{det}\left(\rho_{j\to i}\right)}{{\left(c+k_{v}X_{0}\right)}^{2}\delta^{2}}\\ & - & \frac{\left(1-m_{\mu }\right)\left(1-n_{\mu }\right)\beta_{\mu }p_{\mu }\rho_{\mu \mu }T_{0}}{\delta (c+k_{v}X_{0})}-\frac{\left(1-m_{\text{wt}}\right)\left(1-n_{\text{wt}}\right)\beta_{\text{wt}}p_{\text{wt}}\rho_{\text{wtwt}}T_{0}}{\delta (c+k_{v}X_{0})}. \end{array}$$

### Implementing stochasticity

The occurrence of a mutation is a discrete, singular, stochastic event, while our model consists of continuous differential equations to determine the amount of virus and the number of cells of each type at a given time. Integration of the differential equations represents the average behaviour of the system. This is particularly problematic when modelling phenomena that occur in small numbers, such as the emergence of drug resistant mutants. The differential equations allow for fractions of virus to infect fractions of cells, so our equations might predict a serious illness when the infection would die out in reality. In order to correct for this averaging, we use a semi-stochastic numerical implementation of the differential equations.

We modify the standard Euler method to advance the solution at each time step [64]. We use the Euler method to determine the values of our variables at each time step. However, if the number of cells or virus is not an integer, we interpret the decimal value as the probability that there is an additional virion or cell. For example, if at some time step the Euler method calculates *V* = 102.67 virions, we interpret this as a 67% chance that there are 103 virions and a 33% chance that there are 102 virions. We draw a random number from a uniform distribution to determine whether there are in fact 102 or 103 virions and propagate that integer value forward to the next time step using the Euler method. Since our primary purpose is to study dynamical differences caused by antiviral mechanisms, surface proteins, and the immune response—effects that should be large compared to the effect of stochasticity—this algorithm provides sufficient stochasticity.

In order to use a discrete, semi-stochastic differential equation, however, we must convert the viral titer measured experimentally in units of TCID_50_/mL of nasal wash (*V*) to the number of infectious virions at the site of infection, *N*_*V*_ = *αV*, where *α* (number of virions/[TCID_50_/mL]) is this conversion factor. *α* gives the number of infectious virus particles in one TCID_50_/mL. This rescaling requires that we also adjust the virus production rate (*p*) and infection rate per virion (*β*) which both carry units of virus, such that *β* → *β*/*α* and *p* → *pα* [10]. It is not entirely clear what the value of *α* should be. Previous estimates suggest that 1 TCID_50_/mL of nasal wash corresponds to 10^2^–10^5^ [38] or 3 × 10^4^–3 × 10^5^ [43] virions at the site of infection.

### Measuring emergence of drug resistance

We examine several measures to characterize the influence of drug-resistant mutants during the infection. The fraction of mutants in the cumulative viral titer is the ratio of total number of drug-resistant mutants produced during the infection to the total amount of virus (both wild-type and mutant) produced during the infection, or more formally,

$$\begin{array}{c} \frac{{\tilde{V}}_{\mu }}{{\tilde{V}}_{\text{total}}}=\frac{\int_{0}^{\infty }V_{\mu }\left(t\right)dt}{\int_{0}^{\infty }\left(V_{\text{wt}}\left(t\right)+V_{\mu }\left(t\right)\right)dt}. \end{array}$$ (5)

The fraction of mutants in the cumulative viral titer provides a measure of the relative contribution of drug-resistant mutants to the infection. We also measure the total number of mutants in the cumulative viral titer, ∑*V*_*μ*_. The number of mutants produced during the infection is related to the probability of transmitting the virus to other individuals [38]—higher numbers of mutants leads to a greater probability of passing on the mutant virus to other people. The last measure we use to characterize the emergence of drug resistance is the time of detection. We define the time of detection as the time at which the drug-resistant mutants cross the threshold of detection (defined as 10 TCID_50_/mL) in an infection initiated with a viral inoculum consisting entirely of wild-type virus. This measure will differentiate between typical rapidly-growing infections and slow-growing infections that are likely to be suppressed by an immune response.

## Results

### Efficacy of antiviral treatment

**Fig 3:**
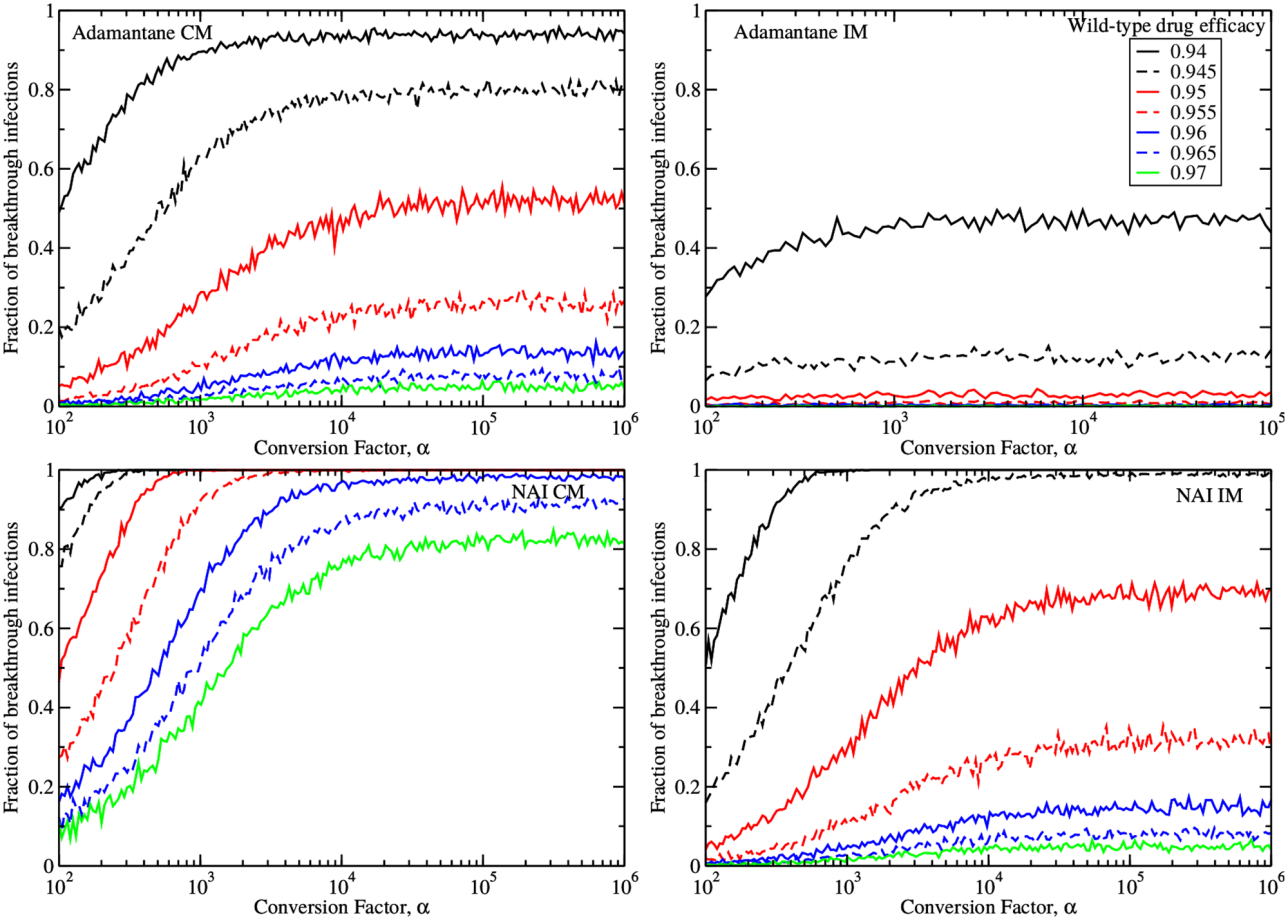
Breakthrough infections. Fractions of simulated infections (out of 1000) resulting in breakthrough symptomatic infections despite treatment initiated at infection onset with either adamantanes (top row) or NAIs (bottom row), assuming the complete (left column) or internal (right column) mutation model as a function of the conversion factor, *α*. Mutant and wild-type virus are assumed to have equal fitness.

Since the conversion factor of virus from TCID_50_/mL to infectious virus particles (*α*) might affect the dynamics of drug resistance, we first assess the impact of *α* by studying the number of breakthrough infections. We simulated 1000 infections treated with an adamantane or a NAI started at *t* = 0, and inoculated with *N*_*V*_(0) = *αV*(0) virions. We determined the fraction of patients who developed a symptomatic infection despite having received preventive antiviral therapy. We defined a symptomatic infection as one in which the viral titer exceeds the symptomatic threshold of 1% of peak untreated viral titer, as defined in [65]. Furthermore, we assumed that both the wild-type and drug-resistant virions are identical (same fitness), differing only in their susceptibility to the antiviral (*m*_*i*_ or *n*_*i*_). Studies suggest that circulating drug-resistant strains tend to have fitness equivalent to the wild-type strain [21, 66, 67]. The effect of fitness will be examined in later sections. Fig 3 shows the number of breakthrough infections in our 1000 simulated treated infections as a function of the conversion factor, *α*, for increasing antiviral efficacies.

When breakthrough infections occurred, the viral load consisted almost exclusively of drug-resistant mutant virus, with wild-type viral titers typically remaining below the symptomatic threshold. When *α* is small, i.e., when there are few virions per measured TCID_50_/mL of nasal wash, the number of breakthrough infections is small. That is because the rate of virion production, *αp*, is small and provides fewer opportunities for resistance to develop. As *α* is increased, the number of breakthrough infections increases until it reaches a maximum value. The production rate is no longer a limiting factor here and the asymptotic value reflects the balance of a decreasing infection rate and an increasing viral inoculum.

The internal mutation model predicts fewer breakthrough infections for a given drug efficacy than the complete mutation model. This is because an internal mutation will only be carried by the core protein, not the surface protein, giving the antiviral a second chance to prevent its spread through the drug-sensitive surface protein. Both the internal and complete mutation models predict adamantanes are better than NAIs at suppressing breakthrough infections due to resistance emergence. This is because adamantanes act before viral replication, unlike NAIs which act afterwards, leaving little opportunity for a mutation to occur. Interestingly, the fraction of breakthrough infections under adamantane therapy given a late mutation is approximately equivalent to that under NAI therapy for an early mutation.

**Fig 4:**
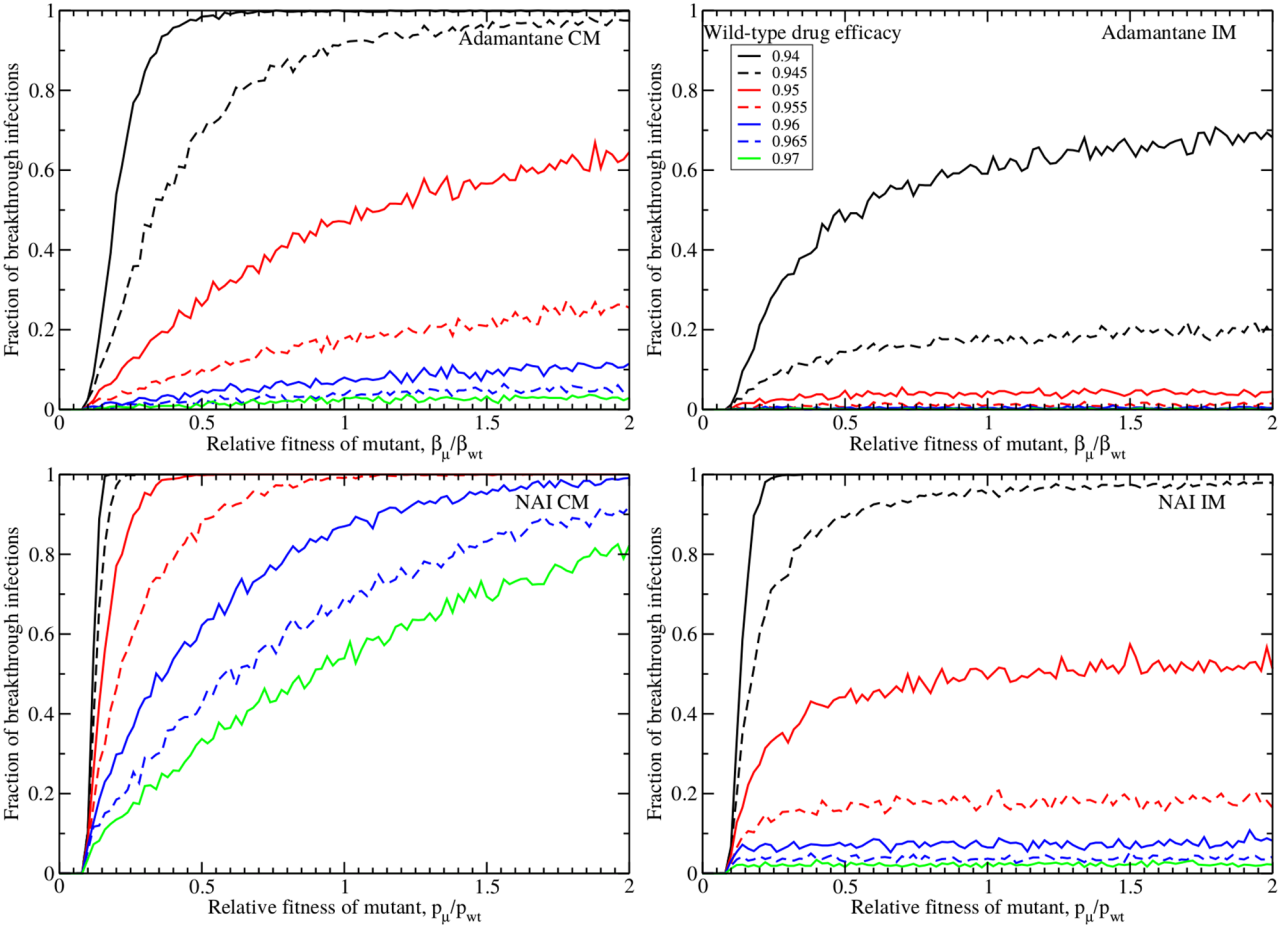
Fitness dependence of breakthrough infections. Number of breakthrough infections during treatment with adamantanes (top row) and NAIs (bottom row) under the complete mutation (left column) and internal mutation (right column) assumptions as a function of the relative fitness of the drug-resistant mutant. The conversion factor is fixed to *α* = 10^4^ virions/[TCID_50_/mL].

To evaluate the effect of the drug-resistant strain’s fitness relative to its wild-type counterpart, we varied the relative fitness while fixing *α* = 10^4^ virions/[TCID_50_/mL], the value at which the fraction of breakthrough infections has reached its asymptotic value and consistent with an estimate of this conversion factor used in [38]. The results are shown in Fig 4. Perhaps the most prominent feature of these plots is the clear fitness threshold needed to produce any breakthrough infections. This threshold is determined by the basic reproductive number of the model, which is the number of secondary infections produced by a single infected cell (see Methods). Essentially, the basic reproductive number must be greater than one in order for the infection to grow and when the fitness of the drug resistant mutant is small, this threshold is not met. When the threshold is surpassed, the number of breakthrough infections rises, sometimes quite dramatically. We again see that for a given drug efficacy, the IM assumption produces fewer breakthrough infections than the CM assumption for both adamantanes and NAIs. Under a particular mutation assumption and for a given fitness and drug efficacy, adamantanes are more effective at suppressing infections than NAIs, but adamantane CM is equivalent to NAI IM.

### Drug resistance in the absence of treatment

Drug resistance has been known to emerge even in the absence of treatment [68]. This is because a mutation which confers resistance against an antiviral almost always emerges over the course of an infection as a result of random mutations [43], but that mutation will only grow to sufficient levels to be detectable in a patient’s viral shedding if its presence does not affect the fitness of the virus significantly. Thus, we set out to determine what conditions will enable a drug-resistant strain to emerge even in the absence of drug pressure.

**Fig 5:**
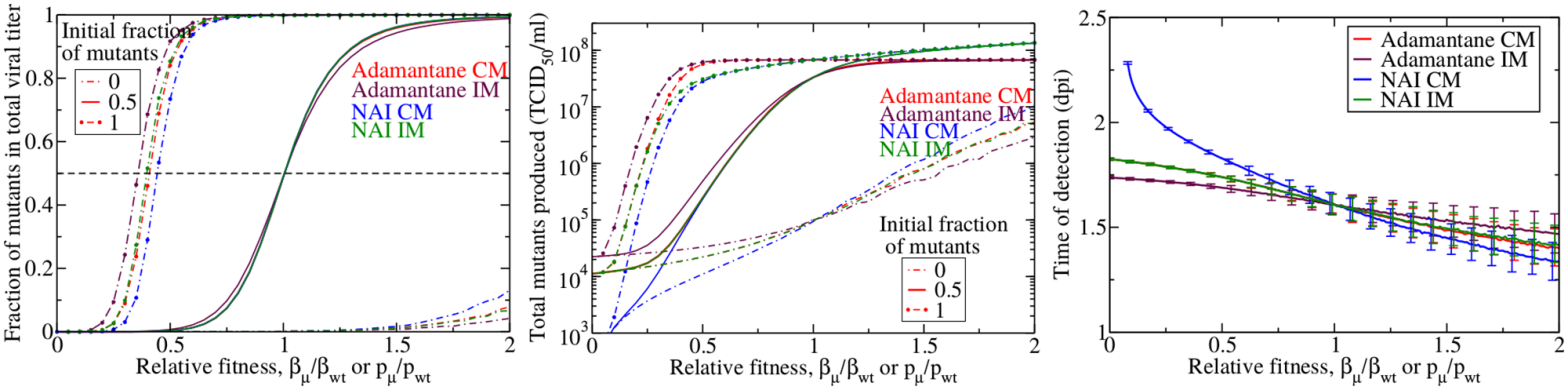
Emergence of drug resistance in the absence of treatment. Fraction (left), total number (center) and time of detection (right) of drug resistant mutants produced in an influenza infection in the absence of drug treatment. Adamantane CM mutants are in red, adamantane IM mutants are in maroon, NAI CM mutants in blue, and NAI IM mutants are in green. We show the mean of 1000 simulations with error bars indicating the standard deviation (error bars are too small to be visible in left two graphs).

Fig 5 shows the three measures of drug resistance described in Methods as a function of the relative fitness of the mutant in the absence of drug treatment. For the fraction of mutants in the cumulative viral titer (left) and the total number of mutants (center), we also investigated the effect of infection initiation with different initial virus mixtures consisting of entirely wild-type, entirely mutant, or a mixture containing 50% of each.

In the absence of drug treatment, the fraction of drug-resistant mutants (Fig 5, left) is negligible when their relative fitness is low (<0.2)—even if the initial viral inoculum consists entirely of drug-resistant mutant. When the viral inoculum consists entirely of wild-type virus, the fraction of drug-resistant mutants remains negligible unless the drug resistant mutants have a high relative fitness. When the initial viral inoculum consists of both wild-type and mutant virus, the infection switches from predominantly wild-type virus to predominantly drug-resistant mutant near a relative fitness of 1. There are, however, some dynamical differences caused by the mutation assumptions and mechanism of drug action. When the initial viral inoculum consists of 100% mutant virus, IM adamantane-resistant mutants become dominant at slightly lower fitness than the other models and the CM NAI-resistant mutants become dominant at slightly higher fitness. The order is reversed when the initial viral inoculum is 100% wild-type virus. In the case of a 100% mutant inoculum, the IM models, which we’ve seen lower the effective rates of mutations, also lower the rate of reverse mutations from mutant to wild-type such that when there are already large numbers of mutant virus, the IM models prevent growth of the wild-type virus. For a particular mutation assumption, adamantanes require a lower fitness to dominate the infection than NAIs when the initial inoculum consists of mutant virus. This is because the fitness cost for adamantane resistance is assumed to affect the infection rate *β* which reduces the chances of infecting a cell, whereas the fitness cost for NAIs is assumed to affect the infection rate *p* which means that the few virions that escape the cell can easily infect new cells and continue the infection.

Even in the absence of drug treatment, we still see the total number of drug resistant mutants produced during the infection (Fig 5, center) rising to very high levels at relative fitness less than 1. The total number of mutants also shows some interesting dynamical differences between the two drug treatments and mutation assumptions. This can be seen particularly at the extremes of viral inoculum (initial inoculum consisting entirely of mutant or entirely of wild-type), where the adamantane-resistant mutants are a slightly larger fraction of the total viral titer than the NAI-resistant mutants at low relative fitness (<0.5), and a slightly smaller fraction of the total viral titer at high relative fitness (>1.5). This difference is due to the small (or large) value of *p*_*μ*_ of NAI-resistant mutants. At low relative fitness, the production of NAI-resistant mutants is suppressed and so NAI-resistant mutants are not detected. As the relative fitness increases, *p*_*μ*_ increases without bound, as does the number of NAI-resistant mutants produced and released over the course of the infection, so the mutants become a large fraction of the population at lower relative fitness than the adamantanes. We also finally see a difference in the dynamics of adamantane CM and NAI IM models. The NAI IM behaves like the adamantane CM model at low relative fitness where the internal mutation incurs an extra fitness cost. At high fitness, however, the few virions with mismatched RNA and surface proteins are far outnumbered by conventionally packed virions, so their effect on infection dynamics is minimal and we see little difference between the IM and CM models for both adamantanes and NAIs.

The time of detection (Fig 5, right) of drug resistant mutants ranges between 1.7–2.3 days post-infection (dpi) at low relative fitness and 1.3–1.5 dpi at high relative fitness. Not unexpectedly, there is a steady decrease in the time of detection as mutant fitness increases because the mutants can spread more easily as their fitness increases and they are therefore detected earlier in the infection. Interestingly, there are differences in the time of detection of NAI-resistant and adamantane-resistant mutants. When the relative fitness is less than 1, the NAI CM mutants are the slowest to emerge because of the drastically reduced production rate of these mutants. NAI IM mutants will not have production suppressed when they are initially produced as they are packed in with the wild-type surface proteins and so emerge a little sooner. The adamantane IM mutants are the fastest to emerge because when initially produced, they have the wild-type surface proteins and so can easily infect cells which will then produce drug-resistant mutants. The situation is reversed once the relative fitness is greater than 1. The NAI CM mutants now have increased production causing them to emerge rapidly and the adamantane AP mutants have a reduced infection rate causing them to emerge later.

### Drug resistance in the presence of treatment

**Fig 6:**
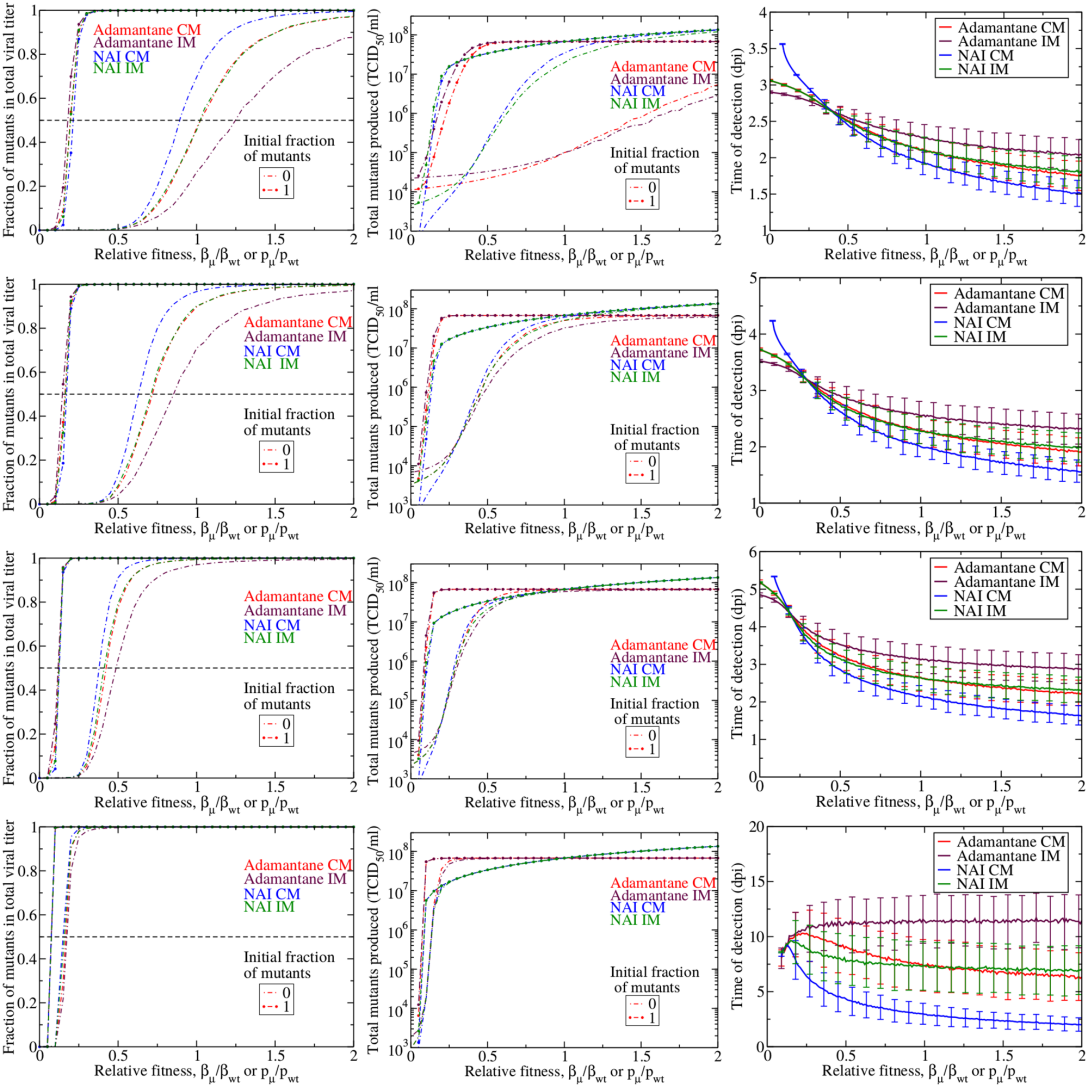
Emergence of drug resistance in treated infections. Fraction (left column), total number (center column) and time of detection (right column) of drug resistant mutants produced in an influenza infection given drug treatment at 60% (top row), 70% (second row), 80% (third row), and 90% (bottom row) efficacy. Adamantane CM mutants are in red, adamantane IM mutants are in maroon, NAI CM mutants in blue, and NAI IM mutants are in green. We show the mean of 1000 simulations with error bars indicating the standard deviation.

In the presence of drug treatment, the drug-resistant mutants have a competitive advantage and are expected to emerge sooner and at lower relative fitness than in the absence of drug treatment. We examine our three measures of drug resistance during treatment initiated at *t* = 0 in order to understand the nature of this competitive advantage. The results are shown in Fig 6, which shows the fraction of mutants in the viral titer (left column), total number of mutants (center column), and time of detection (right column) for infections treated at 60% (top row), 70% (second row), 80% (third row), and 90% (bottom row) efficacy.

In the presence of drug treatment, the relative fitness at which drug-resistant mutants begin to dominate the infection shifts to lower relative fitness. Not surprisingly, drug resistant mutants rise to high levels at low fitness in the presence of drug treatment. We still see dynamical differences between the two drug treatments and the two mutation models, particularly for infections initiated with an inoculum consisting entirely of wild-type virus. These differences, however, diminish as the drug efficacy increases. It seems that the competitive advantage conferred by high efficacy drug treatment trumps any small additional competitive advantage incurred by drug mechanism or viral surface proteins.

Perhaps more important than the number of mutants produced is the time at which the mutants will become detectable during the infection. As the drug efficacy increases, the time to detection of mutants also increases. While this seems somewhat contradictory in the face of the competitive advantage of the drug-resistant mutants, we must remember that we are considering infections initiated entirely with wild-type virus. As the drug efficacy increases, the growth rate of the wild-type infection slows and it takes longer to produce that first drug-resistant mutant. Once that first mutant appears, it has the competitive edge and will grow quickly, but it is the long wait for that first mutant virus that increases the mean time to detection. Unfortunately, this highlights a potential problem with our model. Slow-growing infections that fester for 5–10 d before producing detectable levels of mutant virus are unlikely to occur in most humans. The human immune response will likely clear a slow-growing infection before it has the opportunity to produce a drug-resistant mutant.

### Delayed treatment

**Fig 7:**
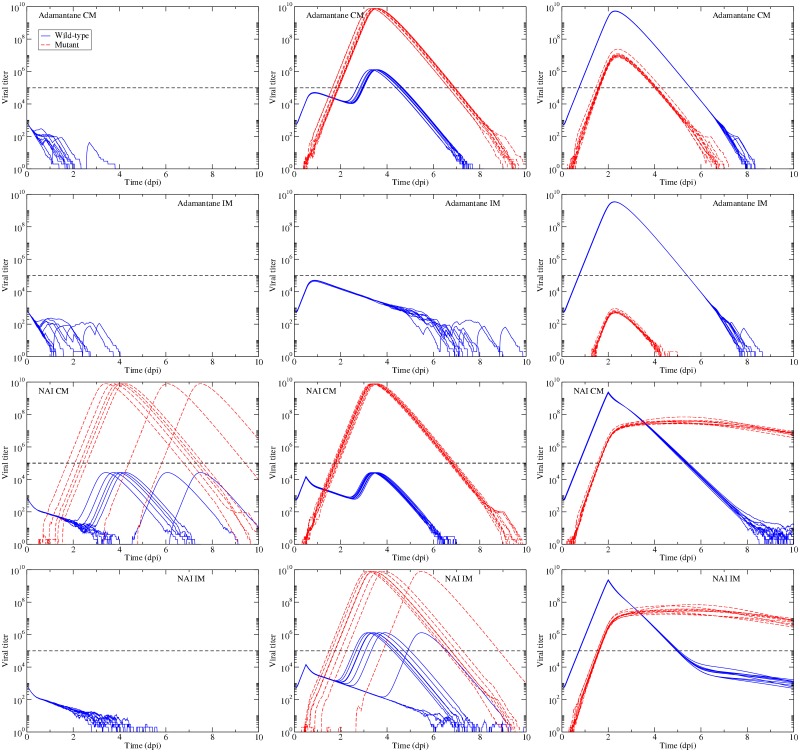
Delayed treatment. Wild-type and mutant virus for treatment initiated at *t* = 0 (left column), at 12 h (center column) and at 48 h right column. The top row shows dynamics for the adamantane CM model; the second row shows the adamantane IM model; the third row shows the NAI CM model; and the fourth row shows the NAI IM model. In each plot, we present 10 simulations. Treatment efficacy is assumed to be 98% and the two strains are assumed to have equal fitness. The dashed line denotes the threshold of detection.

Another short-coming of the previous section is the assumption of treatment initiated at the onset of infection. While both adamantanes and NAIs are occasionally used prophylactically to prevent influenza outbreaks from spreading, they are more often given to patients who are not only already infected, but are most likely experiencing symptoms [69]. The delay in treatment allows the wild-type virus to grow unimpeded for some time affording the opportunity for a drug-resistant mutant to arise stochastically in the absence of antiviral pressure. Initially, this drug resistant mutant will not have a competitive edge, but once antiviral therapy begins, whatever drug-resistant mutants have been produced suddenly have a competitive advantage and will begin to grow. In order to determine what effect delayed treatment might have on the emergence of drug-resistant mutants, we simulated infections with treatment at 98% efficacy initiated at 0, 12 and 48 h post-infection. The results are shown in Fig 7.

An efficacy of 98% is sufficient to suppress all breakthrough infections in three of the four models when applied at the onset of infection. Even with a 12 h delay, the adamantane IM model predicts suppression of the infection, while the remaining models predict that there will be breakthrough infections. The only model for which there is no risk to treatment is the adamantane IM model, since even treatment delayed by 48 h does not result in mutant virus rising to detectable levels. For the remaining models, treatment, while potentially beneficial to the patient, presents the risk of encouraging a drug-resistant infection. This is particularly evident for the adamantane CM and NAI IM models which predict that treatment initiated at *t* = 0 will suppress infections, but when treatment is delayed by even as little as 12 h, which is still before symptoms usually appear, many patients will develop drug-resistant infections. Another potential risk is seen in the predictions made by the two NAI models, which show long-lasting drug-resistant infections when treatment is initiated at 48 h.

### Effect of an immune response

**Fig 8:**
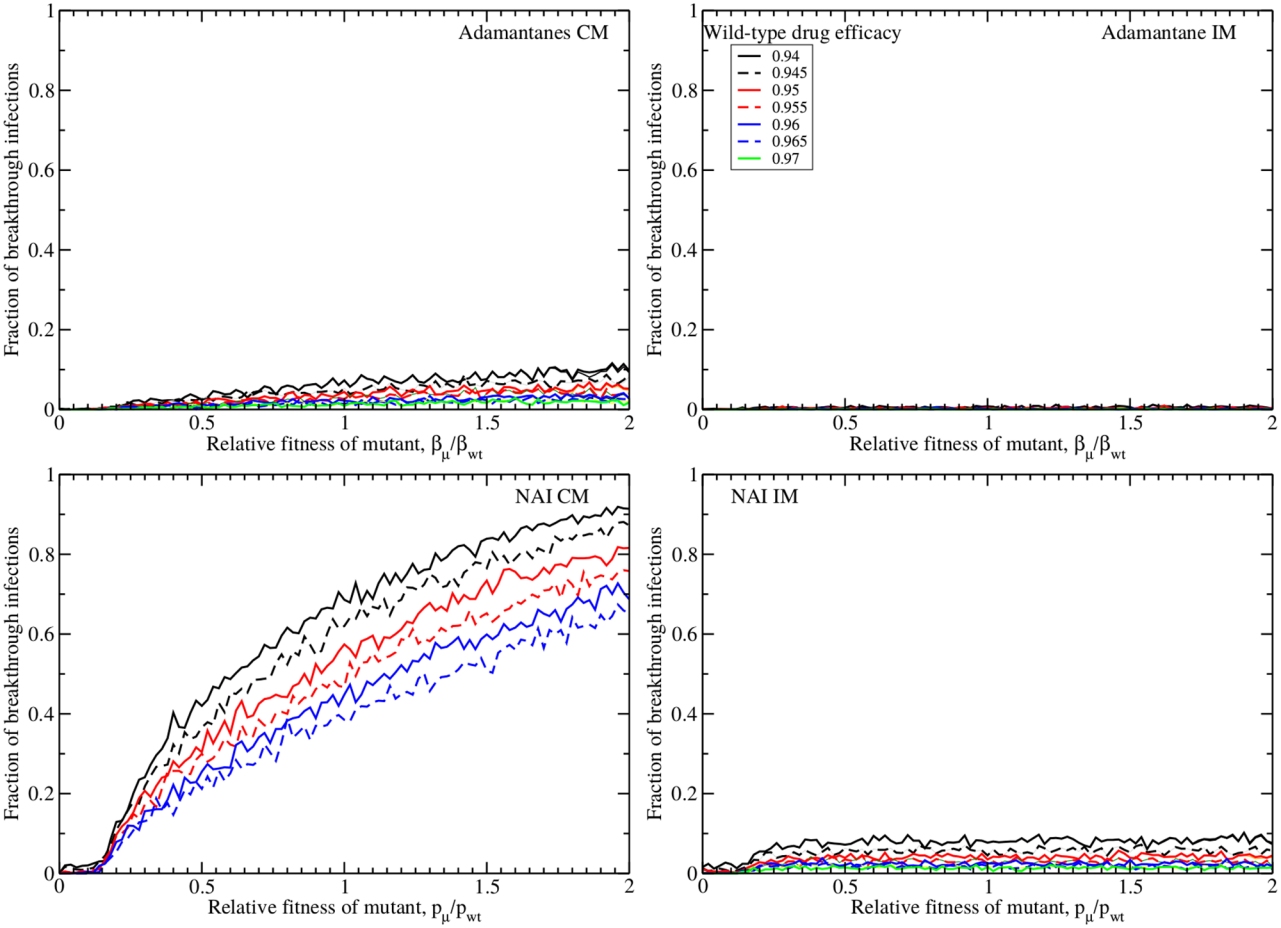
Reduction of breakthrough infections by the immune response. Number of breakthrough infections in the presence of an immune response during treatment started at *t* = 0 with adamantanes (top row) and NAIs (bottom row) under the complete mutation (left column) and internal mutation (right column) assumptions as a function of the relative fitness of the drug-resistant mutant. The conversion factor, *α* is fixed to 10^4^.

To evaluate the effect of an immune response, we first examine the number of breakthrough infections (Fig 8). When we use the same drug efficacies as for the model without an immune response, we see that for all models except the NAI CP, the number of breakthrough infections falls to less than 10%. Even for NAI CP, the number of breakthrough infections is reduced in the presence of an immune response, although it still remains quite high—about 50% of treated patients will become symptomatically infected when the mutant fitness is equal to the wild-type fitness. We again see that there is a minimum fitness needed to produce breakthrough infections. Since the immune response has changed the basic reproductive number of our model, the minimum fitness threshold for breakthrough infections to occur is slightly higher than in the absence of an immune response (∼15% with the immune response compared to ∼10% without).

**Fig 9:**
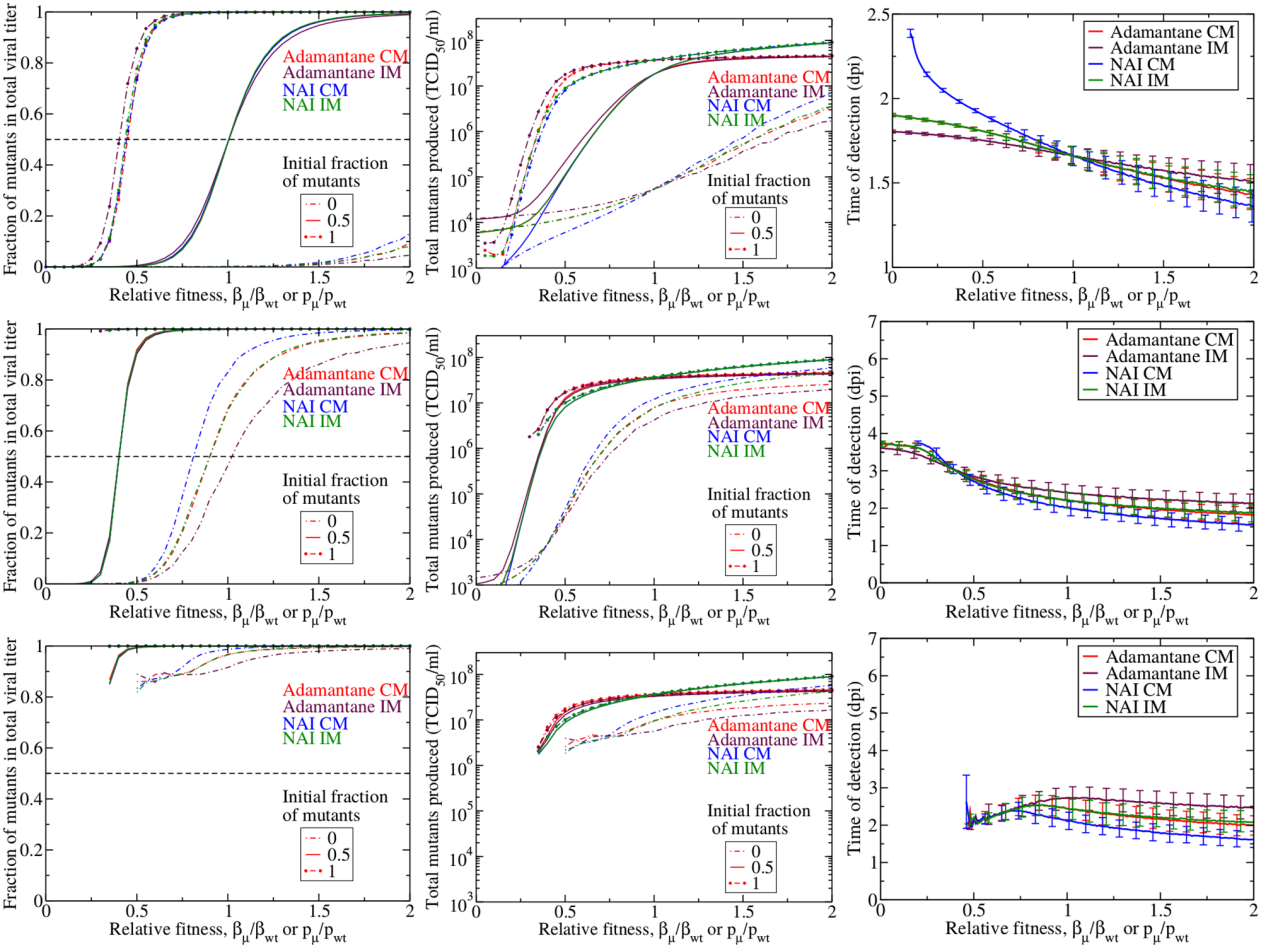
Effect of the immune response on emergence of drug resistance. Fraction (left column), total number (center column) and time of detection (right column) of drug resistant mutants produced in an influenza infection in the presence of an immune response given no drug treatment (top row) and drug treatment at 60% (second row) or 70% (bottom row). Adamantane CM mutants are in red, adamantane IM mutants are in maroon, NAI CM mutants in blue, and NAI IM mutants are in green. We show the mean of 1000 simulations with error bars indicating the standard deviation.

The addition of an immune response also alters the infection dynamics both in the presence and absence of drug treatment. Fig 9 shows the fraction of mutants, the total number of mutants and the time of detection for infections in the absence of treatment (top row) and in the presence of treatment at 60% (center row) and 70% (bottom row) drug efficacy. In the absence of drug treatment, the fraction of mutants, total number of mutants and time of detection in the presence of an immune response look quite similar to those found without the immune response (Fig 5). Careful inspection, however shows that the mutants need a slightly higher fitness to dominate the infection and will take slightly longer to reach detection levels when an immune response is present. When there is an immune response, the mutants need to not only successfully compete for resources with the wild-type virus, but they also need to evade the immune response making it even harder for them to multiply. In the presence of drug treatment, the effect of the immune response is more evident. With 60% drug efficacy, the mutants must have a relative fitness of at least 0.2 in order to produce an infection; with a 70% drug efficacy, the mutants need to have a relative fitness of at least 0.5 in order to produce an infection. This minimum threshold was not observed in the absence of an immune response because mutant virus could linger and slowly grow over long periods of time, so that even mutant virus with very low fitness could eventually multiply to high numbers. The immune response puts an end to these slow-growing infections so that if a mutant virus is not fit enough to multiply to large numbers before the immune response kicks in, there will not be an infection. Since these slow-growing infections have been eliminated, the time of detection is substantially reduced in the presence of drug treatment, with the average time of detection remaining below 4 dpi.

## Discussion

While this is a modelling study, some of the predictions of our models have important implications that warrant further investigation. Our results indicate that it is particularly crucial to prevent breakthrough infections as they contain a large fraction of drug-resistant mutants which can then be spread to other people. Our models indicate that there is a minimum relative mutant fitness relative to the wild-type strain below which there will be no breakthrough infections. For the particular parameters used in our model, this threshold is quite low (mutants need only be 10% as fit as the wild-type virus in the absence of an immune response and about 15% in the presence of an immune response) and is determined by the basic reproductive number. Beyond this threshold, the fraction of breakthrough infections rises sharply as the fitness increases, and reaches an asymptotic value that is determined by the drug efficacy. The lower the drug efficacy, the higher the number of breakthrough infections. This is particularly concerning because there is some evidence that the efficacy of oseltamivir, the most commonly stockpiled antiviral [70], can be fairly low (30–80%) [42, 71]. Amantadine also appears to have a somewhat low efficacy *in vitro*, between 50–95% [10, 72], although this low value could be partly due to the emergence of drug-resistant mutants over the course of the infection [10, 12]. A further exacerbating factor in humans is the person-to-person variability of drug efficacy caused by individuals’ variability in pharmacokinetic parameters [73]. This variability means that some people receiving the standard drug regimen will be receiving treatment at low efficacies, increasing the possibility of a breakthrough infection. The immune response helps to mitigate some of these problems, lowering the drug efficacy at which breakthrough infections rise to high levels. Unfortunately, even with the immune response, the typical efficacy of antivirals is below the efficacy needed to suppress breakthrough infections. These results suggest that early or preventative antiviral therapy is a risky proposition and that it might contribute to the emergence and spread of drug resistant mutants.

Even in the absence of any drug treatment, our models predict that drug-resistant mutants will be present during an infection, potentially in large enough numbers to be transmitted to other individuals. We have the following potential scenario for the spread of drug-resistant mutants even in the absence of treatment. An individual is infected with 100% wild-type virus, and even though the drug-resistant mutant has low relative fitness (let’s say 0.5) and remains a small fraction of the cumulative viral titer, there are still ∼10^4^ TCID_50_/mL of drug resistant mutants produced over the course of the infection. The number of mutants becomes detectable in the patient’s nasal wash somewhere between day 1.5–2 post-infection, fairly early in the infection. This patient, then, is shedding a detectable amount of drug-resistant mutant for some part of his illness. This individual infects a second individual, but now the viral inoculum contains a small fraction of drug-resistant mutants. The second individual now produces a slightly larger fraction of mutants in their infection and passes that larger fraction on to the next individual. In this way, drug-resistant mutants can propagate within the population even in the absence of the selective pressure of drug treatment. This finding suggests that some evidence of drug resistance, albeit possibly at low levels, is expected in the population even in the absence of any drug treatment within the population. This was the case for both amantadine and oseltamivir, where drug-resistant strains persisted in the population at low levels for years in spite of the absence of widespread antiviral treatment [4, 14]. Since we don’t see high levels of drug resistance in the absence of treatment, there must be mechanisms suppressing growth and transmission of the drug resistant variants. This could be due to low fitness of the drug resistant mutants [25, 26], or the effect of the immune response, both of which would lower the amount of drug resistant virus produced during an infection. Studies also indicate that some drug resistance mutations can also cause impairment of transmission fitness [74–76], although this is not the case for all drug resistance mutations [23, 75–78].

During treatment, the drug-resistant mutant has a competitive edge and rises to high levels at lower fitness than in untreated infections. When drug treatment is delayed, the wild-type virus has some opportunity to grow and possibly produce drug-resistant mutants such that once drug treatment is applied, the drug-resistant mutant will be able to grow without interference or competition from the wild-type virus. We found that even a treatment delay as short as 12 h could result in predominantly drug-resistant infections. This is particularly problematic because typical treatment delays are 36–48 h since people will not seek treatment until after they start to experience symptoms. This longer treatment delay also leads to possibly risky scenarios, at least for NAIs, where we see that initiation of treatment at 48 h produces a long-lasting (>10 d) drug-resistant infection, which provides a longer window for spread of the drug-resistant virus to other people. Thus, delayed treatment can also encourage the growth and spread of drug-resistant virus.

Previous work has estimated the total number of virions as well as the number of single nucleotide mutants produced during an uncomplicated infection [43], estimating that the total number of virions is between 10^10^ and 10^12^ and the number of single nucleotide mutants is on the order of 10^10^. Our models provide a similar estimate, finding that the total number of virions produced is on the order of 10^8^ TCID_50_/mL or, using our conversion factor of *α* = 10^4^ virions/[TCID_50_/mL], on the order of 10^12^ virions, consistent with the previous estimate. For low relative fitness, our models predict 10^8^–10^9^ drug-resistant mutants. This is lower than the previous estimate since they estimated the number of virions with a single nucleotide mutation at any location, whereas our work counts the number of virions with a single nucleotide mutation at a specific location. Since the number of nucleotides in the influenza genome is 1.4 × 10^4^, the previous estimate suggests that there should be on the order of 10^6^ mutants with mutations at a specific location, which is lower than our estimate. The main reason for the difference between these two estimates is due to the assumed nucleotide mutation rate (7.3 × 10^−5^ per nucleotide per replication used here versus 2 × 10^−6^ per nucleotide per replication used in [43]), so we expect that our model will predict a higher number of mutants.

Beyond these general findings for the emergence of drug-resistant mutants, our model has helped elucidate differences between the emergence of adamantane-resistant mutants and NAI-resistant mutants. In the presence of drug treatment, NAI-resistant mutants emerge faster and at lower relative fitness than adamantane-resistant mutants. The dynamical differences we see are because the two drugs act at different points in the replication cycle. NAIs act at the end of the replication cycle, they prevent the production and release of susceptible virions [5, 6], but do not interfere with virion entry and RNA replication processes. This means that the wild-type virus can get into the cell easily and can replicate its RNA, potentially generating a drug-resistant mutant. While the wild-type virus cannot leave the cell due to the effect of the NAI, the drug-resistant mutant can escape the cell, allowing NAI-resistant mutants to dominate the population very rapidly. Adamantanes, on the other hand, act at the beginning of the replication cycle, blocking infection of the cell [2]. In this case, the wild-type virus cannot even enter the cell to begin replication and does not have a chance to produce a drug-resistant mutant. Thus, if all else (fitness, drug efficacy, etc.) is equal, it is better in terms of preventing drug resistance to treat influenza with adamantanes or some antiviral that acts at the beginning of the replication cycle, than with NAIs or an antiviral that acts at the end of the replication cycle. This finding should be considered as researchers develop new antivirals for influenza, with antivirals acting early in the replication cycle being preferable to those acting later, at least from the standpoint of mitigating drug resistance.

Our model also considered the effect of virion external packing on the emergence of drug resistant mutants. The internal mutation model, which assumes that there is a mismatch between the viral surface proteins and the RNA contained within for newly mutated strains, essentially gives drugs a second chance to prevent the drug-resistant mutant from spreading. When the drug-resistant mutant is initially created, its RNA is packed in a capsid that contains the wild-type surface proteins which are sensitive to the drug and will prevent spread of the mutant. The wild-type exterior, then, acts as a further hindrance to the emergence of drug resistance in the presence of drug treatment. Dynamical differences between IM and CM models are evident even in the absence of drugs, although it tends to be less dramatic here. In this case, the differences are due to the decrease (or increase) of fitness of the surface proteins. In reality, there is likely to be a mix of surface proteins on the surface of virions created from a new mutation, although this has not been experimentally examined. Many parts of the viral replication cycle are still not clearly understood [79], with bud formation and release being among the least understood processes [80]. In order to develop accurate models of influenza infection, however, the details of the replication process need to be thoroughly understood. As our models show, sometimes the details of the process can dramatically affect infection kinetics.

While there are other modeling studies of the emergence of drug resistance in the literature, this study does provide new insights. A study by Canini et al. canini14 uses viral kinetic modeling to examine emergence of drug resistance during oseltamivir treatment in both immunocompetent and immunocompromised patients. Studies by Loverdo et al. [81, 82] do not specifically examine drug resistance, but do examine the impact of early versus late mutations on emergence of mutants. Neither of these papers examine the effect of antiviral mechanism of action. While amantadine is no longer really in use due to widespread resistance [4], our finding of differences in emergence of drug resistance for antivirals that act early in the replication cycle versus those that act late in the cycle will also be true for new antivirals. Our results, then, will help researchers understand the potential for drug resistance in new antivirals. This is not, however, the only novel insight in this paper. While some aspects of the paper have been considered separately, this paper examines all of them together, providing insight into how the different effects interact. For example, while the Loverdo papers examine the effect of surface proteins, they do not consider the effect of drug pressure or the immune response. Similarly, the Canini paper does not include the effects of surface proteins or antiviral mechanism. In addition, our study uses endpoints not considered in the other studies, such as the number of breakthrough infections and the time of emergence, and examines them as a function of viral fitness, a factor not considered in the previous studies. This allowed us to identify a threshold fitness needed for emergence of drug resistant mutants, an effect not observed in previous studies. Thus, our study provides a more complete picture of the factors that affect the emergence of drug resistance.

Since most of our results examine emergence as a function of viral fitness, it is rather difficult to experimentally verify some of the specific model predictions. First, while our model assumptions allow for a simple definition of relative fitness, in practice there are several quantities that can be used to characterize viral fitness [53, 54]. If we found that one of these measures of fitness corresponds well to the relative fitness used in our model, we would then need to systematically alter the fitness of a virus, or identify several strains of virus with different fitness in order to verify the model predictions. However, it is possible to experimentally verify whether amantadine or NAI treatment lead to different amounts of drug resistant mutants being produced or whether the time of detection changes with different antivirals for a particular strain of influenza.

Even though we included several biological processes not included in previous mathematical models of drug resistance, our models are still simplified representations of the infection process. Our inclusion of an immune response was simplistic, including only antibodies, even though other components such as interferons and cytotoxic T-lymphocytes will also help to clear the infection [63]. Interferon does not directly clear virus, and while it shortens the infection, typically by protecting some target cells from infection resulting in fewer cycles of infection, it is unable to clear an infection [63, 83]. Interferon also lowers viral load, so we expect that fewer virions will be produced during the infection, although we would expect the proportion of drug-resistant to wild-type virus to be the same since interferon acts on cells, not virus, and should affect both strains equally. The time of detection might be delayed in the presence of an innate response since viral titer is lowered, meaning that it will take longer for virus to grow to detectable levels. Cytotoxic T-lymphocytes clear infected cells, but in experiments, they have a similar effect on viral titer as the antibody response [63], so we expect that including this immune response would not substantially change our results. We also neglected the pharmacokinetics of the antivirals and assumed that drug efficacy remained constant throughout the infection, although a recent study suggests that a constant drug is a good approximation when studying viral titers [84]. Finally, our study did not examine patient-to-patient variability, as was done in a previous study [42], and its role in the emergence of drug resistance.

## Conclusion

Our models present a simplified version of viral dynamics which help to elucidate some of the mechanisms driving the emergence of drug-resistant mutants during the course of a single infection. Our models show that the mechanism of drug action as well as the surface proteins present on the surface of the mutant virus both play a role in how quickly drug-resistant mutants will emerge and how many mutants will be produced over the course of the infection.
